# How Should We Grade the Quality of a Transthoracic Echocardiogram: Results from a Survey from the Association for European Pediatric and Congenital Cardiology (AEPC) Imaging Working Group

**DOI:** 10.1007/s00246-025-03914-5

**Published:** 2025-06-10

**Authors:** Inga Voges, Massimiliano Cantinotti, Owen Miller, Gerald Greil, Heynric Grotenhuis, Almudena Ortiz-Garrido, Francesca Raimondi, Colin J. McMahon

**Affiliations:** 1 Department of Congenital Heart Disease and Pediatric Cardiology, University Hospital Schleswig-Holstein, Kiel, Germany; 2 DZHK (German Centre for Cardiovascular Research), Partner Site Hamburg/Kiel/Lübeck, Kiel, Germany; 3 Department of Pediatric Cardiology and Congenital Heart Disease, National Research Council-Tuscany Region G. Monasterio Foundation (FTGM), Massa, Pisa, Italy; 4 Evelina London Children’s Hospital, London, UK; 5 University of Texas Southwestern (UTSW)/Children’s Health, 1935 Medical District Drive, Dallas, TX 75235 USA; 6 Department of Pediatric Cardiology, Wilhelmina Children’s Hospital/UMCU, Utrecht, The Netherlands; 7 Paediatric Division, Paediatric Cardiology Department, Hospital Materno Infantil, Regional Universitario de Málaga, Málaga, Spain; 8 Paediatric Cardiology Department, John Paul XXIII Children’s Hospital, Bergamo, Italy; 9 Department of Paediatric Cardiology, Children’s Health Ireland at Crumlin, Dublin 12, Ireland; 10 School of Medicine, University College Dublin, Belfield, Dublin 4, Ireland; 11 Maastricht School of Health Professions Education, Maastricht, The Netherlands

**Keywords:** Pediatric echocardiography, Quality criteria, Feedback, Training, Transfer of training

## Abstract

The aim of this study was to assess the current status about quality grading, resources, and training in pediatric echocardiography, to define gaps in this field, and to develop potential strategies for quality improvement. A structured questionnaire was sent out to pediatric cardiologists within the Association for European Pediatric and Congenital Cardiology (AEPC). The questionnaire contained questions regarding assessment of quality, training and feedback in the field of pediatric echocardiography. Thirty-one European pediatric cardiologists from 17 countries participated. Most participants agreed (*n* = 28, 90%) that it is important to have standards for echocardiography quality grading for trainees. Objective instruments, however, are largely not available. Among a list of criteria on how to grade quality, quantitative or qualitative criteria with additional formative feedback was ranked highest by the respondents (53%). Although the correct diagnosis, followed by the correct use of two-dimensional (2D) imaging and the correct use of color Doppler across all valves and septae were listed as most important when performing transthoracic echocardiography, a matrix of the eight most important parameters was designed. The results show that quality grading in pediatric echocardiography varies highly among European centers. The matrix provided is a visual instrument whereby trainees can gauge the evolution in their skill as echocardiographers.

**Supplementary Information:**

The online version contains supplementary material available at 10.1007/s00246-025-03914-5.

## Introduction

Echocardiography represents the first-line cardiovascular imaging modality in pediatric cardiology and has dramatically improved the field together with other imaging modalities since its beginning in the last century. Although performance and reporting standards for pediatric echocardiograms have been published [1, 2], very limited information exists regarding the quality criteria in pediatric echocardiography and specifically how to grade the quality of pediatric transthoracic echocardiograms. Levine et al. reported on competency testing of pediatric cardiology fellows as meets expectations, fails to meet expectations, or exceeds expectations, but this remains a relatively subjective assessment [3].

Since pediatric cardiology covers a wide range of age groups and diseases including patients with normal anatomical and functional findings up to those with complex cardiovascular anatomies (e.g., heterotaxy syndromes, functional single ventricle), pediatric echocardiography is a challenging and demanding technique for which specialist training and standardization is mandatory [1, 4–7].

Although this is widely acknowledged, only a few studies have addressed quality aspects of pediatric echocardiograms, the impact of resources and training and how to reduce diagnostic errors [8–14]. Published studies that clearly describe the knowledge gap and provide approaches for standardized quality improvement are lacking. Nevertheless, it is important to have a standard of quality for assessment of transthoracic echocardiography for quality improvement and training of congenital cardiology trainees to ensure that all information is gathered and correct diagnoses are made.

In this study, we used a structured questionnaire to (1) describe the current quality grading, resources and training in pediatric echocardiography in European pediatric cardiology departments, to (2) describe current knowledge gaps regarding quality aspects, and to (3) provide an overview of how quality grading can be improved.

## Methods

### Structured Questionnaire

A structured questionnaire was sent out by email to fully trained pediatric cardiologists in Europe who are members of the Association for European Pediatric and Congenital Cardiology (AEPC) Imaging Working Group (IWG). The survey was generated by two pediatric cardiologists and validated by the committee of the IWG made up of six pediatric cardiologists. The survey was comprehensive, including 36 questions (Table 1).

**Table 1:** Questions and answer options of the survey

Questions	Answer options
I am currently employed as a …	– General paediatric cardiologist
	– Imaging paediatric cardiologist with primary role in echocardiography
	– Imaging paediatric cardiologist performing echo, MRI, fetal
	– Other role
	– Other (please specify)
The country I work in is …	Name of the country
The center I work at is in …	Name of the center
The number of transthoracic echocardiograms performed per year at our center is …	Number of transthoracic echocardiograms
Our center trains young doctors in paediatric cardiology including echocardiography	Yes/No
It is important to have some standard by which to judge the quality of echocardiograms for trainees	– Strongly agree
	– Agree
	– Neither agree nor disagree
	– Disagree
	– Strongly disagree
	– Other (please specify)
A more sophisticated evaluation instrument for transthoracic paediatric echocardiography is needed (national/international core laboratory) than what is currently available	– Strongly agree
	– Agree
	– Neither agree nor disagree
	– Disagree
	– Strongly disagree
	– Other (please specify)
There are adequate objective instruments currently available to judge the quality of a transthoracic echocardiogram performed by a fellow	– Strongly agree
	– Agree
	– Neither agree nor disagree
	– Disagree
	– Strongly disagree
	– Other (please specify)
There are only subjective measures to evaluate the quality of a transthoracic echocardiogram	– Strongly agree
	– Agree
	– Neither agree nor disagree
	– Disagree
	– Strongly disagree
	– Other (please specify)
Rate the following in importance in performing an echocardiogram	Answers—please see Fig. 2
Which of the following are critical in performing a competent echocardiogram (must be performed well)?	Answers—please see Fig. 3
How would you best grade each of these criteria?	– Qualitative (poor, average, good, excellent)
	– Qualitative (meets expectations, fails to meet expectations, exceed expectations)
	– Quantitative (0–3 Poor, 4–5 Fair, 6–7 Good, 8–9 Very good, 10 Excellent)
	– Quantitative (percentile 0–25, 26–50, 51–75, 76–100)
	– One of the above with additional formative feedback (verbal or written)
	– Other
	– Other (please specify)
Echocardiogram report should be first generated by the trainee and reviewed by trainer (supervisor)	– Strongly agree
	– Agree
	– Neither agree nor disagree
	– Disagree
	– Strongly disagree
	– Other (please specify)
Rank which aspects of the echocardiogram report are most important	– Correct diagnosis
	– Correct segmental anatomy description
	– Quantification of AV valve function
	– Quantification of semilunar valve function
	– Ventricular function (M-mode, Ejection fraction)
	– *Z*-scores for all relevant structures
	– Other
Would an instrument to monitor trainee progress in echocardiography based on the above parameters help with training?	– Strongly agree
	– Agree
	– Neither agree nor disagree
	– Disagree
	– Strongly disagree
	– Other (please specify)
How soon do fellows echo on their own after starting training?	– Less than 1 month
	– 1–3 months
	– 3–6 months
	– > 6 months
	– When they demonstrate competence
	– Other (please specify)
Feedback to fellows is critical for them to improve their echo skills	– Strongly agree
	– Agree
	– Neither agree nor disagree
	– Disagree
	– Strongly disagree
	– Other (please specify
How do you provide feedback to trainees on their echo skills/standard?	– Formative (description of what is good and what could be better)
	– Summative (pass/fail)
	– Combination of above
	– Other
	– Other (please specify)
Feedback is best provided during the fellows performing echocardiogram and allowing them to optimize their imaging skills in real time under trainer supervision	– Strongly agree
	– Agree
	– Neither agree nor disagree
	– Disagree
	– Strongly disagree
	– Other (please specify
Encouraging fellows to present their echocardiogram during the multidisciplinary conference (surgical and cardiology conference) facilitates their learning and should be encouraged.	– Strongly agree
	– Agree
	– Neither agree nor disagree
	– Disagree
	– Strongly disagree
	– Other (please specify)
Rate in your opinion the most important factors (most important at top) in promoting transfer of training in transthoracic echocardiography for trainees	Answers—please see Fig. 6
I am satisfied with how we train our trainees in echocardiography	– Strongly agree
	– Agree
	– Neither agree nor disagree
	– Disagree
	– Strongly disagree
	– Other (please specify
When initiating echocardiography training does the trainee scan with a cardiologist/sonographer?	Yes/No
Trainees starting transthoracic echocardiography training image normal hearts and basic lesions before advancing to more complex cases	– Strongly agree
	– Agree
	– Neither agree nor disagree
	– Disagree
	– Strongly disagree
	– Other (please specify
What barriers are challenging in training fellows in echocardiography	Answers—please see Fig. 7
Do you have a curriculum for transthoracic echocardiography training at your center?	Yes/No
Is transoesophageal echocardiography part of the echocardiography training in your center?	Yes/No
Do you provide training in advanced echocardiographic techniques and post-processing measurements (e.g., 3D and strain measurements)?	Yes/No
Do you provide simulation in echocardiography in your center?	Yes/No
Is there a separate specialist track for training in fetal cardiac imaging?	Yes/No
Trainees should be trained in use of 3D echocardiography when indicated?	– Strongly agree
	– Agree
	– Neither agree nor disagree
	– Disagree
	– Strongly disagree
	– Other (please specify
Trainees should be trained in use of stress/strain imaging when indicated?	– Strongly agree
	– Agree
	– Neither agree nor disagree
	– Disagree
	– Strongly disagree
	– Other (please specify
How can we train trainees to image cardiac lesions they have not previously encountered (i.e., transfer of training)?	– Free answers—please see Supplement 1
What more can we do to promote training in echocardiography for trainees: What more would you like to see to foster training young doctors in echocardiography?	– Free answers—please see Supplement 1

The questionnaire aimed to describe current approaches in how the quality of pediatric echocardiograms are judged. More specifically, the questionnaire included questions about the importance of quality criteria, the grading and performance of echocardiograms, training and feedback as well as the use of more advanced techniques and resources (please see Table 1).

### Statistical Analysis

Statistical analysis was performed using MedCalc® (MedCalc Software Ltd, version 22.021). Data are expressed as numbers (*n*) and percentages (%).

## Results

### Participants

**Fig. 1:**
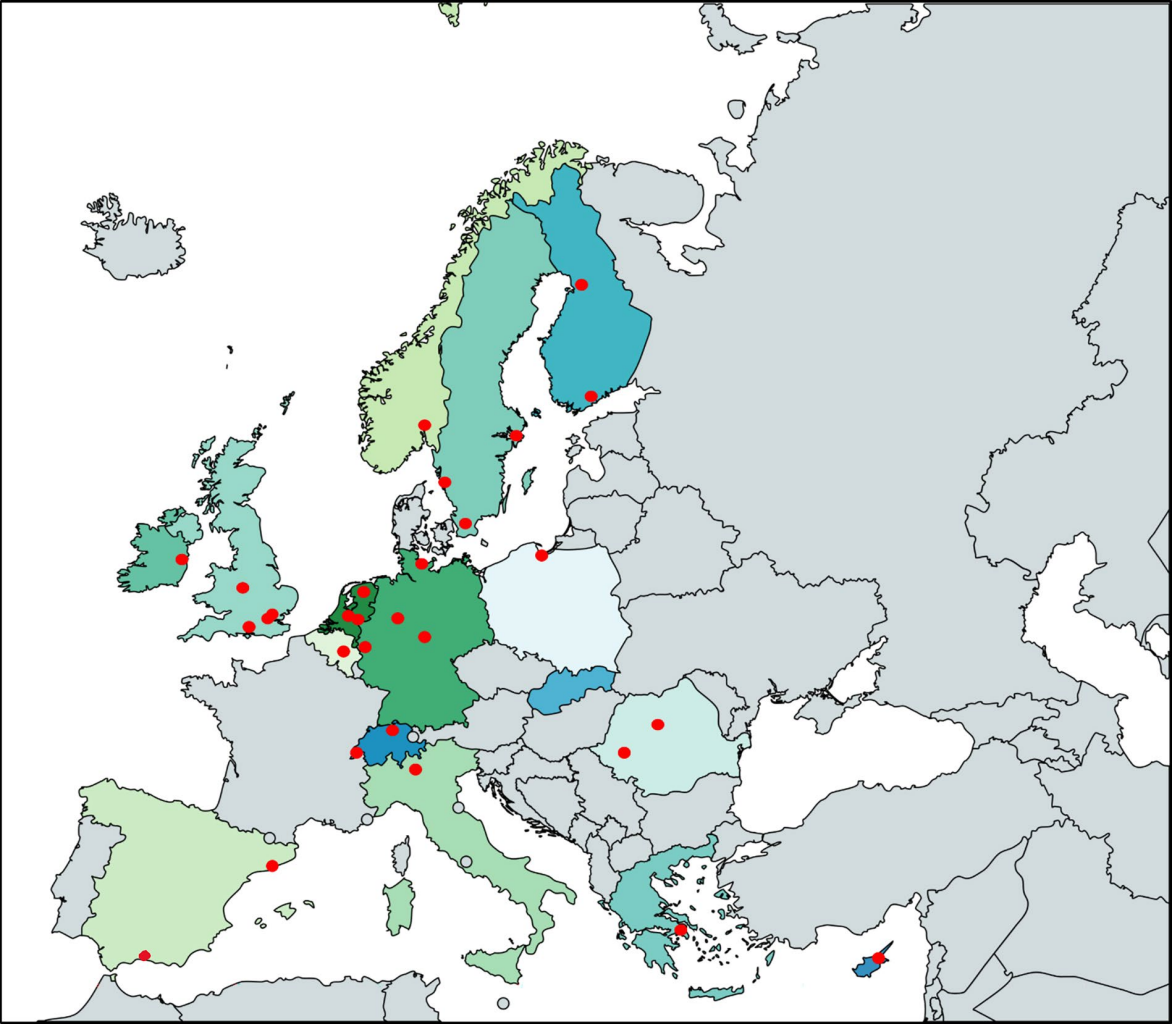
Countries and centers that participated in this survey. Thirty-one centers from 17 countries participated in the study

Thirty-one pediatric cardiologists from different centers and from 17 countries responded to the invitation and gave consent to participate in this study (Fig. 1). Types of employment with imaging specialties of the study participants are shown in Table 2. The number of transthoracic echocardiograms (TTE) in each participating center ranged from 1200 to 20,000 per year.

**Table 2:** Type of employment and imaging specialties

Characteristics of study participants (*n* = 31)	
Profession • General pediatric cardiologists • Imaging pediatric cardiologists (echocardiography) Imaging pediatric cardiologists (echocardiography, MRI and fetal imaging) • Other roles (alone or in addition) – Imaging pediatric cardiologist (echocardiography, MRI) (*n* = 1) – Imaging pediatric cardiologist (echocardiography, fetal echocardiography, and CT) (*n* = 1) – Director of a congenital cardiology unit (*n* = 1) – Professor in pediatric cardiology (*n* = 1)	32.3% 32.3% 32.3% 12.9%

### Instruments to Measure TTE Quality

Most survey participants strongly agreed (*n* = 19, 61.3%) or agreed (*n* = 9, 29%) that it is important to have some standard (core laboratory) to judge the quality of echocardiograms by trainees. In addition, most participants strongly agreed (*n* = 13, 41.9%) or agreed (*n* = 11, 35.5%) that a more sophisticated evaluation instrument (national/international core laboratory) than what is currently available for TTE is needed. Four contributors neither agreed nor disagreed (12.9%) and 3 (9.7%) disagreed with this question.

The question whether adequate objective instruments to judge the quality of a transthoracic echocardiogram performed by a trainee are currently available was answered with ‘agree’ by 10 participants (33.3%).

16 (51.6%) Respondents agreed and 2 (6.5%) strongly agreed that there are only subjective measures to evaluate the quality of a transthoracic echocardiogram.

### What is Important When Performing TTE and How to Grade the Quality?

**Fig. 2:**
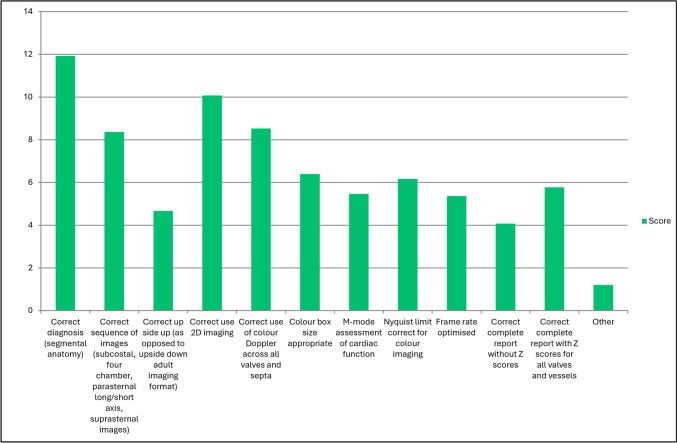
Question 11: rate the following in importance in performing a transthoracic echocardiogram

**Fig. 3:**
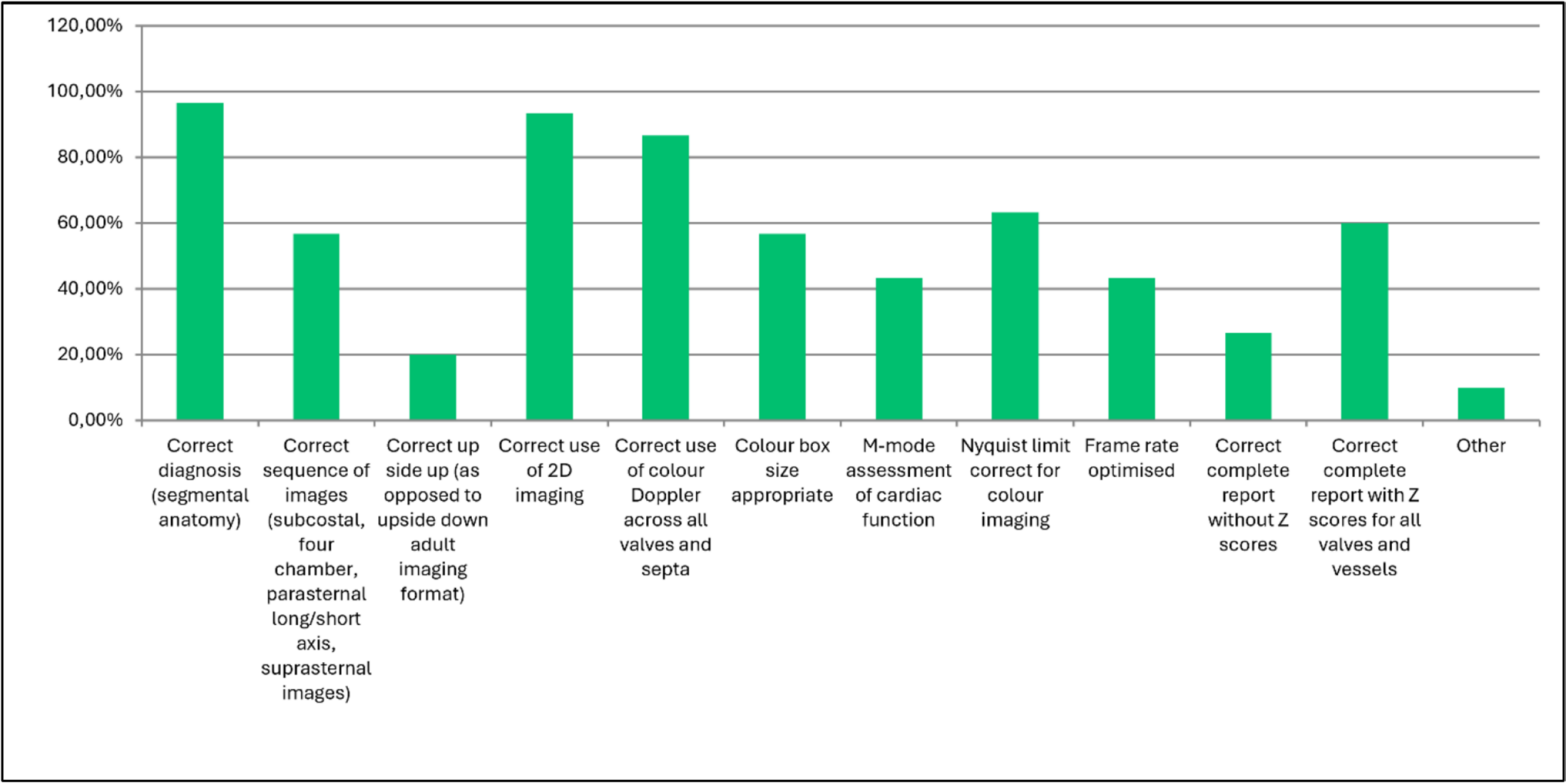
Question 12: which of the following are critical in performing a competent echocardiogram (must be performed well)? Responses are showing as percentages

The most important from a list of response options when performing TTE was found to be the correct diagnosis followed by the correct use of two-dimensional (2D) imaging and the correct use of color Doppler across all valves and septae (Figs. 2, 3; Table 1).

**Fig. 4:**
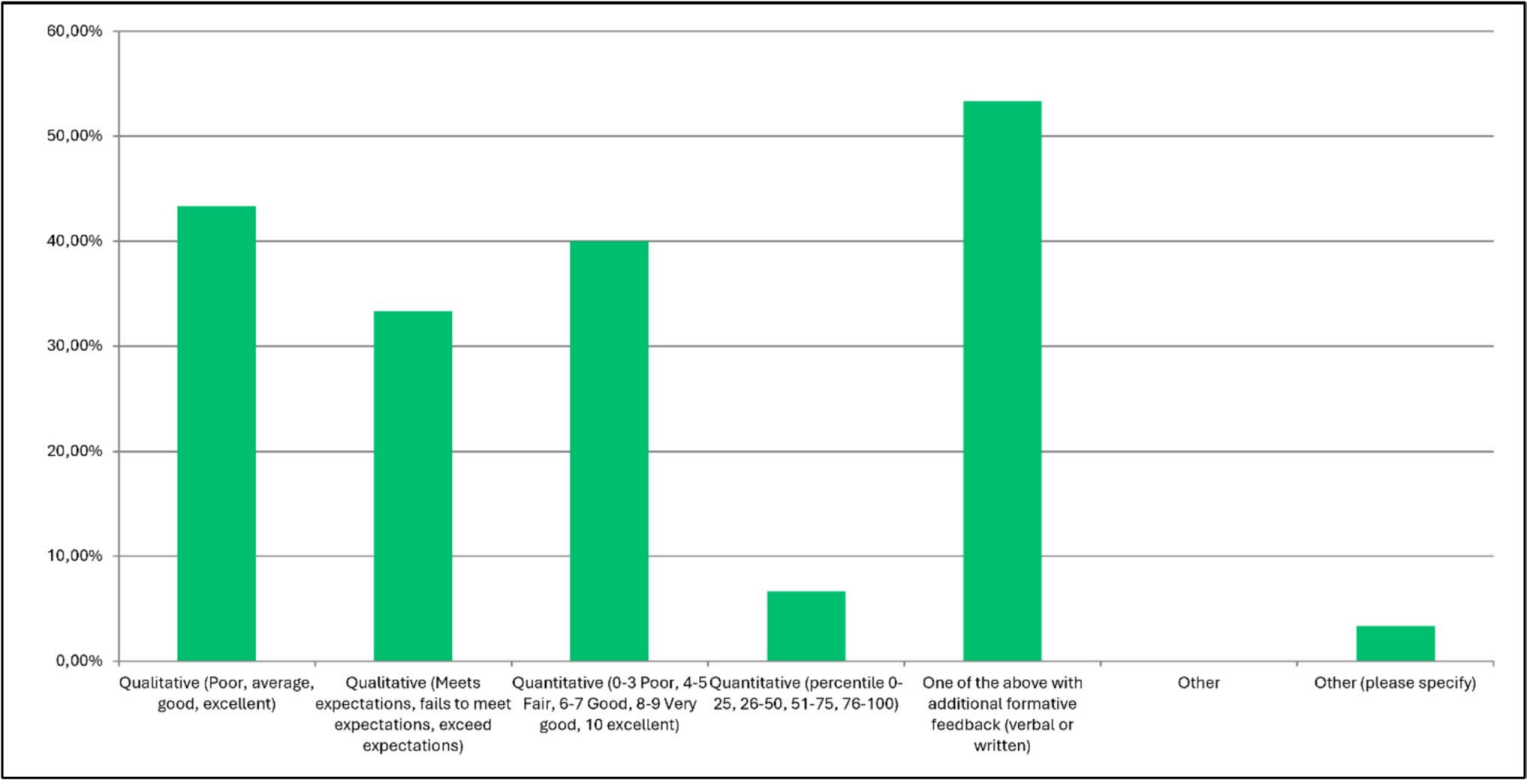
Question 13: how would you best grade each of these criteria (please pick 2 best ones)?

The participants were asked which two criteria out of a provided list (see Table 1) they would find best describe the quality of a TTE. Quantitative or qualitative criteria with additional formative feedback (verbal or written) were ranked highest by the respondents (53.3%) followed by qualitative only (poor, average, good, excellent; 43.3%), quantitative only (0–3 poor, 4–5 fair, 6–7 good, 8–9 very good, 10 excellent) and quantitative described by percentiles (percentile 0–25, 26–50, 51–75, 76–100) (see Fig. 4). One respondent commented that the quality grading should include the question whether the TTE answers the clinical question.

#### Visual Matrix of Performance

A matrix (illustrative Fig. 8, see also Supplement 2) which graded eight key parameters in performing and interpreting echocardiography is provided. Each parameter is scored from 0 to 5 with 0 not performed, 1 poorly performed, 2 adequately performed to be interpreted, 3 well performed (reaches competence expected of the novice), 4 performed very well, and 5 performed to an excellent level (level expected of an expert).

### Reporting

**Fig. 5:**
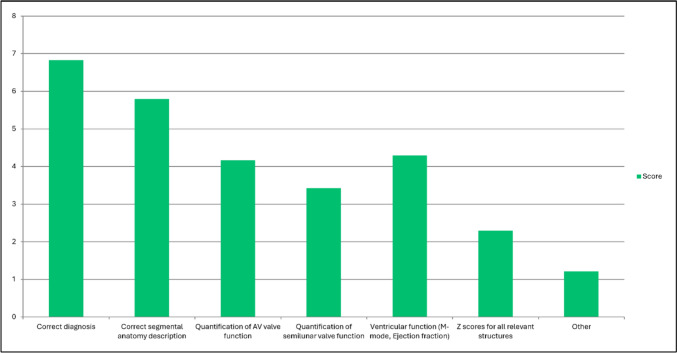
Question 15: rank which aspects of the echocardiogram report are most important

Most participants agreed that an echocardiogram should be first performed by the trainee and then reviewed by the expert trainer (strong agreement, 58.1%; agreement, 38.7%). Reporting elements (Table 3) were ranked regarding their importance. The correct diagnosis reached the highest score, followed by correct segmental anatomy description and ventricular function (M-mode, ejection fraction) (Fig. 5). Quantification of AV valve function, quantification of semilunar valve function, and *Z*-scores for all relevant structures reached lower score values. 43% (*n* = 13) Strongly agreed and 53.3% (n = 16) agreed that an instrument to monitor trainee progress that is based on these reporting aspects can help with training.

**Table 3:** Reporting elements

Reporting element
Correct diagnosis
Correct segmental anatomic description
Quantification of AV valve function
Quantification of semilunar valve function
Ventricular function (M-mode, Ejection fraction)
*Z*-scores for all relevant structures
Other

### Training and Feedback

In 29 participating centers (93.5%) training in pediatric cardiology included training in echocardiography. Two participants (6.5%) reported that pediatric cardiology training is not offered in their center.

Twenty-two participants (71%) were satisfied with how training is offered in their institution. 16% Neither agreed nor disagreed with this question, and 12.9% either disagreed or strongly disagreed. Written responses suggested structured hands-on training with feedback, an echocardiography curriculum, more time for training, and better training assessment tools could be helpful. An echocardiography curriculum for training is available in 43.3% of the participant centers, whereas 56.7% of participants do not have a curriculum.

**Fig. 6:**
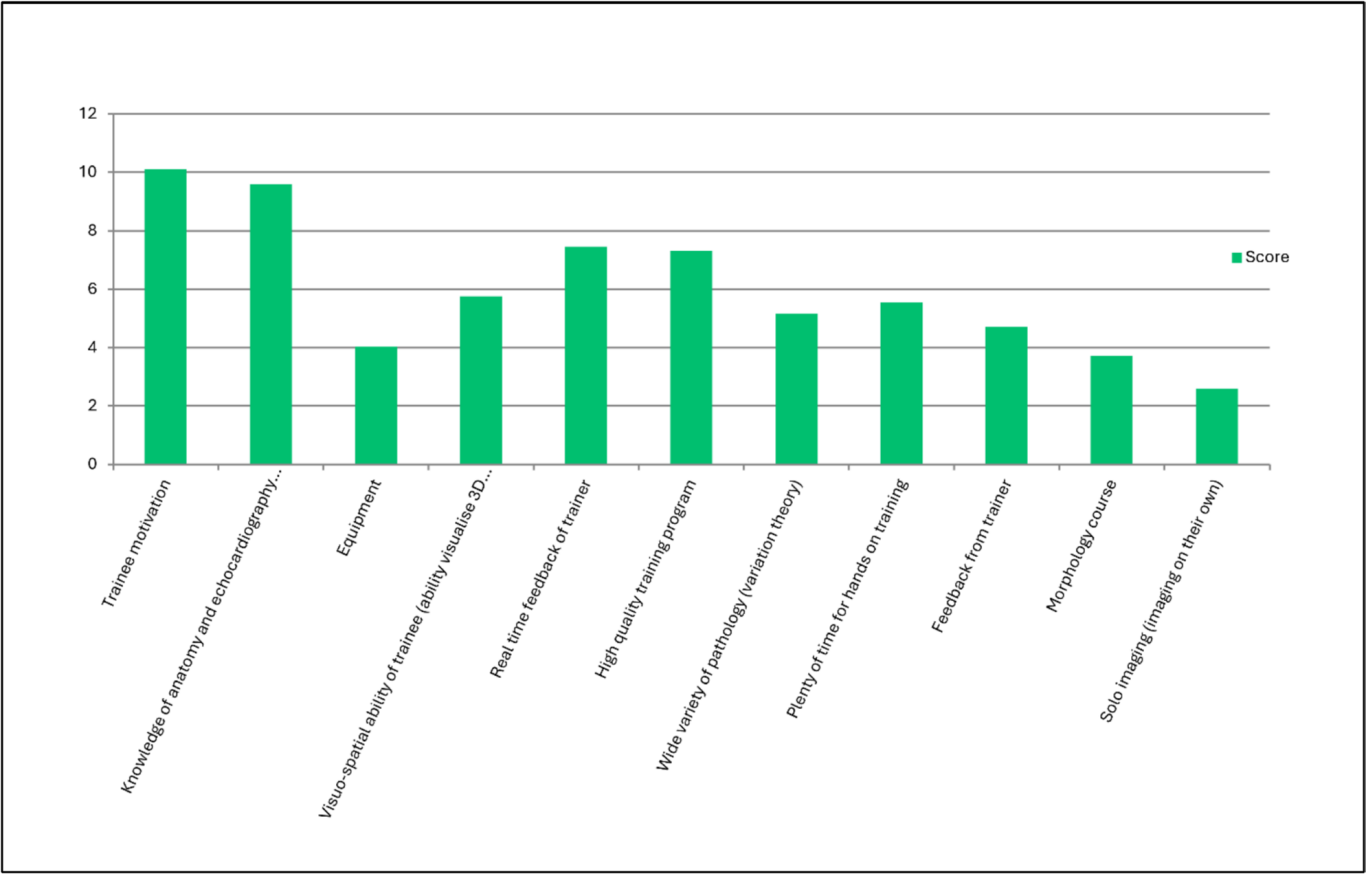
Question 22: rate in your opinion the most important factors (most important at top) in promoting transfer of training in transthoracic echocardiography for trainees

**Fig. 7:**
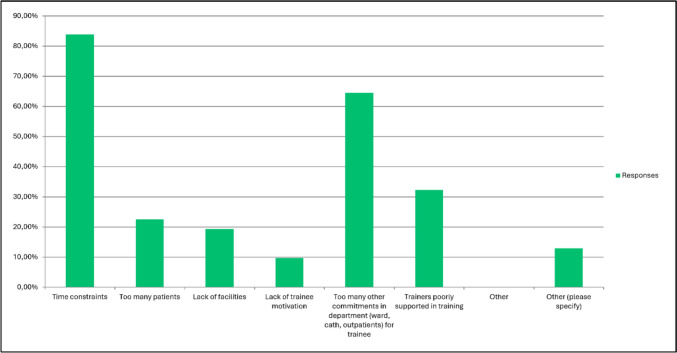
Question 26: what barriers are challenging in training fellows in echocardiography. Responses are showing as percentages?

According to the survey results, the majority of trainees scan together with a cardiologist or sonographer at the beginning of training (93.5%). Trainees commonly start performing echocardiography independently in the first 3 months of training in 54.8%, < 1 month after starting training in 16.1%, and 1 to 3 months after starting training in 38.7%. Seven survey participants (22.6%) answered that trainees perform echocardiography independently when they show competence and 1 (3.2%) responded that they start after more than 6 months. The three most important factors for promoting independent practice were trainee motivation, knowledge of anatomy, and real-time feedback of the trainer (Fig. 6). The question whether trainees should receive a very structured training for the first 3 months with imaging of normal hearts and basic lesions first before advancing to more complex cases was positively answered by 26 participants (86.7%). Barriers for training fellows were mainly related to time constraints (Fig. 7).

There was general agreement that feedback is essential for trainees to improve their skills (strong agreement 87.1%, agreement 12.9%). Most often, feedback is given in a formative format (80%, 24 participants) or as a combination of formative and summative elements (10%, 3 participants). Regarding the timing of the feedback, 87% of study participants strongly agreed or agreed that feedback is best provided during the echocardiogram.

Presenting echocardiographic studies at surgical and cardiology hospital conferences can be part of echocardiographic training, and all respondents strongly agreed or agreed that this facilitates the trainee’s learning and should be encouraged.

### Training in Transoesophageal Echocardiography and Advanced Techniques

Training in transoesophageal echocardiography (TOE) is performed in 58% of the participating centers. 10 Survey participants responded that they do not perform TOE training (32.2%). Written comments included that there is not enough TOE training, that it is only done occasionally in the operating theater, or only for fellows who will stay in the center or those who express interest in becoming an imaging cardiologist.

Training in advanced echocardiographic techniques and post-processing measurements occurs in 51.6% of the centers. The written comments included that advanced techniques are offered when trainees show interest or want to stay in the training center.

Most respondents strongly agreed or agreed (71%, *n* = 22) that trainees should be trained in 3D echocardiography when indicated. Training in strain and stress imaging is also commonly offered (strong agreement /agreement: 83.9%).

### Simulation in Echocardiography

Thirty-two percent of the participants answered that echo simulators are provided, whereas 64.5% said that simulators for echocardiography are not available.

### Fetal Echocardiography

A specialist track for training in fetal cardiac imaging is available in 54.8% of the participating centers.

### Transfer of Training and How to Promote Training

Transfer of training means the extent to which the skills and knowledge learned can be applied in other clinical situations.

Written suggestions are shown in Supplement 1. Trainee motivation, trainee knowledge of cardiac anatomy and echocardiography, and real-time feedback provided to the trainee from the trainer were the three most important factors listed that promote transfer of training for trainees.

## Discussion

Echocardiography is the workhorse in pediatric cardiology and is essential for the diagnosis, management, and long-term follow-up of pediatric patients with congenital or acquired heart disease. Involving AEPC cardiologists was relevant for this study, as they have a global view on recommendations and standards. Nevertheless, the relatively small number of participating European centers restricted the ability to perform statistical subgroup analyses, and the fact that only AEPC members participated introduced a potential selection bias.

We could demonstrate that quality grading, resources, and training in pediatric echocardiography varies highly among European centers. Echocardiography training was provided in most participating centers, but results show that a more standardized training and sophisticated quality grading instruments are needed. Furthermore, this study showed that feedback for trainees is important, but that the feedback methodology is not homogeneous, and a structured and scientifically validated approach is lacking. Training in advanced techniques, TOE, and fetal echocardiography is often not available.

### Gaps in Quality Assessment

Quality grading of pediatric transthoracic echocardiography is important. Although general recommendations for performing echocardiography in children and adolescents exist [1, 2, 15], objective instruments and standardized approaches on how to grade an echocardiogram are largely not available, and approved scoring systems have not yet been established. Only a few studies about this topic exist. Balasubramanian et al. developed a pediatric echocardiography complexity score that allows for better categorization of echocardiographic examinations which in turn might be helpful in allocating resources and trainee education [8, 16]. Hill et al. developed a score and assessed echo lab and patient features that may impact image quality [17]. Another group showed that standardized protocols for pediatric cardiology fellows can improve imaging and reporting [18, 19]. Similarly, a standardized Doppler protocol together with a scoring system was developed and evaluated to improve quality of pediatric echocardiograms [20]. Some studies focused only on certain settings and diseases [21–23], including intraoperative assessments [24]. Others in turn have referenced modern techniques, such as artificial intelligence or simulations [25, 26]. Simulation-based training can improve echocardiographic performance and image quality and may be complementary to other training options [27, 28]. Similar results have been shown for newer echocardiography training programs such as boot camps that have been combined with online learning [29, 30]. Training programs with validated and accredited exams are stimulating education in pediatric echocardiography and can hereby improve quality [31].

Non-pediatric studies have shown that collaboration in a large echocardiographic registry database together with quality improvement pathways are able to improve the completeness of reporting of key echo quality measures [32]. It has also been shown that certification/accreditation can lead to improved comprehensiveness and completeness of echocardiographic studies and reports [33]. A sonographer-led service could improve the quality of pediatric echocardiograms and the management of echocardiographic data, as shown in previous studies [34]. However, sonographers are not available in many European countries due to resources and history [34].

However, a comprehensive quality scoring system for echocardiographic studies does not exist.

### How Can Quality Grading Be Improved

**Fig. 8:**
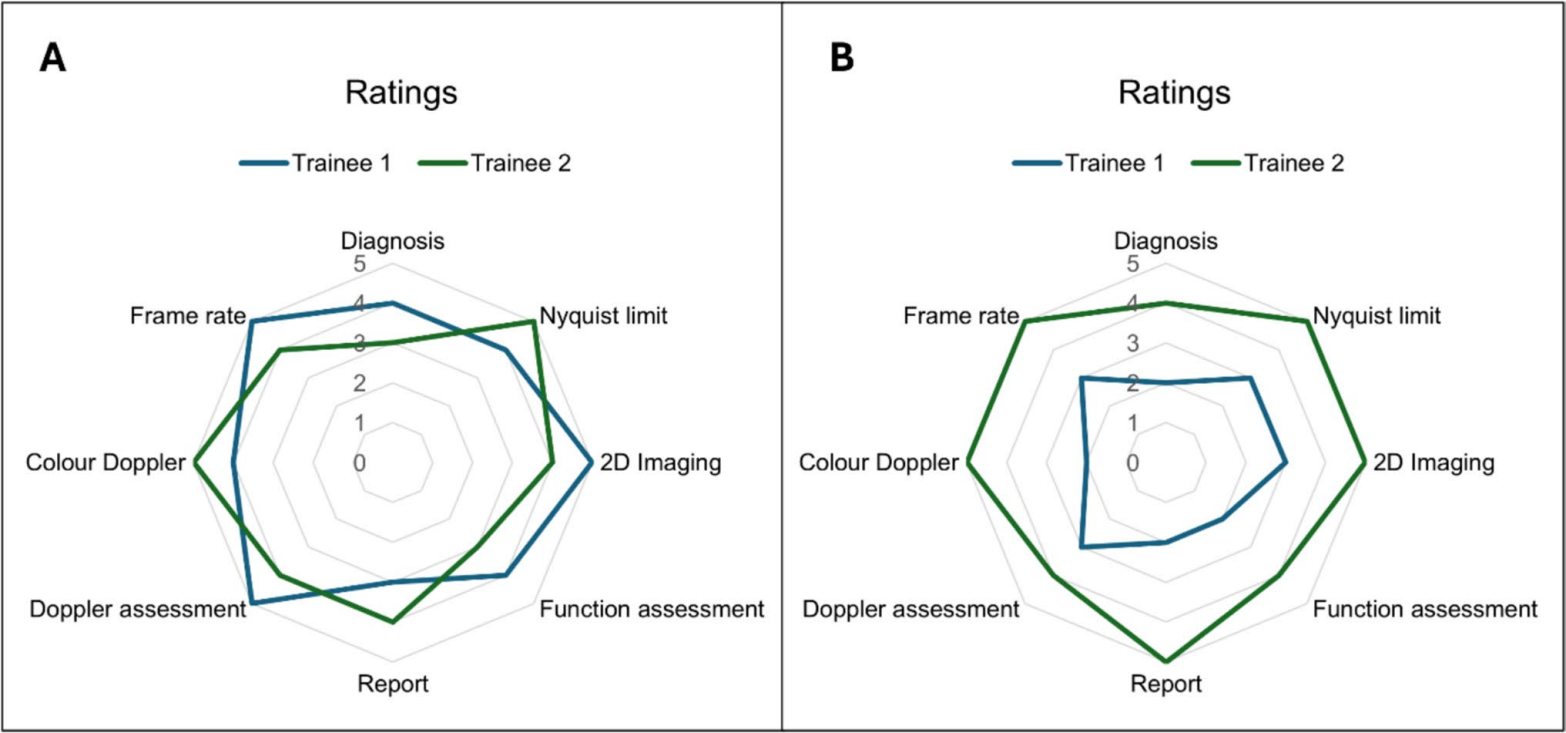
Performance rating of trainees in pediatric transthoracic echocardiography using a visual matrix of performance. Eight parameters (diagnosis, Nyquist limit, 2D imaging, function assessment, report, Doppler assessment, color Doppler, frame rate) are rated, and each parameter is scored from 0 to 5 [0 not performed, 1 poorly performed, 2 adequately performed to be interpreted, 3 well performed (reaches competence expected of the novice), 4 performed very well, and 5 performed to an excellent level (level expected of an expert)]. Example **A** and **B** show ratings for 2 trainees. In **A**, both trainees show good to excellent performance. In **B**, trainee 1 does not reach the score 3 for all parameters, whereas trainee 2 shows very good to excellent performance. Areas for improvement for trainee 3 are diagnosis, function assessment, report, and color Doppler

We propose a visual instrument in the style of a radar chart by which trainees can gauge the progression in their skillset in undertaking transthoracic echocardiography (Fig. 8). This enables the trainee to focus on individual components of their imaging skills which can be improved. The visualization of the matrix of performance allows the trainee to monitor progression over time. An additional self-assessment tool for trainees as well as anonymous feedback from trainees together with new ideas to avoid bias from cardiologists who have developed the training program, can add value.

The authors’ next steps are to conduct pilot testing of the performance matrix, followed by its implementation in echocardiography training for pediatric cardiology trainees at European training centers and to document their feedback. This feedback will inform future recommendations regarding the matrix’s wider use.

### Limitations

Not all respondents answered all questions, and the written answers may represent subjective opinions. Furthermore, the number of participants was relatively small, which restricts the ability to perform regional comparisons and to analyze whether the variation in responses reflects differences in respondent characteristics. As the survey was only sent to members of the AEPC, a potential selection and representative bias cannot be excluded as opinions from other trainers were not included. Our quality assessment tool has not yet been validated in a clinical setting, and objective performance data are not presented, although we plan to test and implement its use to ascertain how trainees benefit from using the matrix instrument in future studies. The scoring system from 0 to 5 for each of the parameters remains somewhat subjective by the trainer assessing the trainee. However, standards for competence and expert performance tend to be reproducible.

## Conclusion

Assessment of pediatric transthoracic echocardiography quality is essential for trainees to gauge their progression in establishing competence as echocardiographers. Furthermore, the matrix provided allows assessment of their progression to independent echocardiographers. This tool will hopefully improve standards of transthoracic echocardiography for trainees and as a consequence, the overall pediatric echocardiography service provided to patients. Nevertheless, structured and validated pediatric echocardiography quality assessment tools, such as this matrix, need to be implemented into training programs to study their efficacy and benefit to trainees.

## Supplementary Information

Below is the link to the electronic supplementary material.

Supplementary file1 (DOCX 21 kb)

Supplementary file2 (XLSX 14 kb)

## Data Availability

No datasets were generated or analysed during the current study.
