# Supplementary material for: How Should We Grade the Quality of a Transthoracic Echocardiogram: Results from a Survey from the Association for European Pediatric and Congenital Cardiology (AEPC) Imaging Working Group
Source: Pediatr Cardiol. 2025 Jun 10;47(3):1354–67. doi: 10.1007/s00246-025-03914-5 (PMC12901096; doi:10.1007/s00246-025-03914-5)
Supplement: Supplementary file 1 — Supplementary file1 (DOCX 21 kb) [file 246_2025_3914_MOESM1_ESM.docx]

**Supplement**

**Supplement Table 1.** Question 34: How can we train trainees to image cardiac lesions they have not previously encountered (i.e. transfer of training)?

| **Answers** |
| --- |
| \| 1. Atlas, simulation, clinical cases archive \| \| --- \| \| 2. By showing them videos \| \| 3. Consistent order of doing things \| \| 4. With formal and unformal teaching. 5. Formal by showing pictures of the lesion so that they can recognise it. Teaching trainees how to image and what are the relevant information’s to get for this lesion. 6. Unformal by providing feedback once they encountered and imaged the lesion. \| \| 7. reviewing images and having a knowledge of morphology \| \| 8. If a trainee is trained to perform echocardiography in a very systematic and structured way (for instance high quality subcostal sweeps) that also helps to image lesions they have not seen before.  9. A good video-based online training platform with clips and a place to share interesting cases could also be helpful maybe? \| \| 10. Simulations \| \| 11. Reviewing previous cases \| \| 12. By a systematic approach \| \| 13. They should call for help if they cannot report, do sweeps over the heart from different angulations \| \| 14. The trainer and trainee should discuss what findings and changes that defect could cause and why \| \| 15. Repository of high-quality video training focusing on specific subjects \| \| 16. To teach sequential segmental analysis \| \| 17. By strict segmental approach 18. By remembering anatomy \| \| 19. We can present images from the echo gallery or using the internet echo library, but for practice, they can also train in different centers, if there is not enough pathology in the center they are enrolled. \| \| 20. Segmental anatomy 21. Formal training \| \| 22. Segmental sequential approach and daily echo review meeting \| \| 23. Facilitate examination of "unusual" and complex cases \| \| 24. Online workshops, central repository of interesting and rare cases (can be center based or a central European database) \| \| 25. Redo echo of such a case already performed by competent paediatric cardiologist or echocardiographer including usual feedback. \| \| 26. Online learning with sending videos of these lesions \| \| 27. Provide pathology echo images and video loops atlas/ archive with theory background or important technical aspects where they can search for the lesion and then look up pathology examples and important technical aspects. \| \| 28. Simulation and banks of echo clips like the UW app \| \| By simulation, by attending courses, by rotation to high volume centres \| \| 29. We have made webinars \| \| 30. Systematic approach. Use of protocols, simulators and web resources. Courses. Subspeciality fellowships at reference centers. \| \| 31. Simulation helps, I think. Also being exposed to a high variation of complexity before helps them work out new cases they have not seen before. Taking segmental approach is critical for them to take an organised sequential approach. allowing them try to work it out themselves I think is important. Then it is vital to give them meaningful feedback on what was good and what could be improved. \| \| 32. Simulations. To increase theoretical knowledge \| |

**Supplement Table 2.** Question 35: What more can we do to promote training in echocardiography for trainees: What more would you like to see to foster training young doctors in echocardiography?

| **Answers** |
| --- |
| \| 1. 3D printed models featuring standard echo crosssections and plains \| \| --- \| \| 2. Technical practicalities associated with the different machines/ vendors \| \| 3. Simulation and morphology courses (lab or virtual reality) are now essential \| \| 4. Make it easy to get hands on from an early in the training. \| \| 5. Promote going abroad and gain experience elsewhere. \| \| 6. More simulation \| \| 7. Trainers with protected time for teaching \| \| 8. We need an official programme to have enough time to dedicate to it \| \| 9. I would need much more time for that! \| \| 10. The trainees’ own motivation is the most important factor. How can we increase that? \| \| 11. Encourage the development of high volume and high-quality training centers that would offer rotations for trainees from smaller centers and countries. \| \| 12. More recognition of the subspecialty expertise required (in my centre it is valued less than intervention) \| \| 13. Train as early as possible 14. Start with the youngest \| \| 15. I think a virtual echo lab is necessary, where they can connect and where they have the possibility to see and learn a lot about echocardiography in paediatric cardiac pathologies and malformations, especially rare cases, that they can see in the practice. \| \| 16. Evidence based training \| \| 17. Echo quality review sessions, nonjudgmental review \| \| 18. Structuralizing the education and hands on as much as possible \| \| 19. An online video crash course (10 hrs +) on basics. We have put together material but would love to see something video based. More frequent AEPC supported based courses. AEPC suggested curriculum. \| \| 20. Use of simulation dolls in the setting of a curriculum. \| \| 21. More time besides clinical workload \| \| 22. More available echo courses and incorporation of morphology \| \| 23. Advocate echolab certification 24. Obligation to be involved in all AEPC fellow courses and examination 25. Review of all training programs available in institutes and learn from best practices \| \| 26. Emphasis on training in educational guidelines, implementation of structured echo protocols as well as training protocols \| \| 27. Simulation training for congenital heart disease is a good option - would there be a possibility to build a product together with some company for this? \| \| 28. Dedicated time with sonographers. Dedicated time with supervising staff. More advanced simulators. Animal studies/research. \| \| 29. More training courses. Greater use of simulation. AEPC do a pretty good job with supporting junior trainees more so with travel bursaries, courses and webinars. \| \| 30. Online courses by AEPC. CME points.  31. Echo conference with interesting cases  32. Review the echocardiographic studies of all fellows together at the end of the day for teaching purposes \| |
